# Mechanical Performance of 3D-Printed Resin Materials for Endocrown Restorations: A Comparative Evaluation of Fracture Resistance, Weibull Analysis, and Experimental Fracture Toughness

**DOI:** 10.3390/jfb17090430

**Published:** 2026-08-26

**Authors:** Osama Abuabboud, Adrian-George Marinescu, Mihai Paven, Izabella-Maria Kovacs, Luminița-Maria Nica, Andrei-Bogdan Faur, Liviu Marșavina, Dan Ioan Stoia, Anca Jivănescu

**Affiliations:** 1 Department of Restorative Dentistry and Endodontics, TADERP Research Center, Faculty of Dentistry, “Victor Babeș” University of Medicine and Pharmacy, 300041 Timișoara, Romania; marinescu.adrian@umft.ro (A.-G.M.); mihai.paven@umft.ro (M.P.); nica.luminita@umft.ro (L.-M.N.); 2 Department of Restorative Dentistry and Endodontics, Faculty of Dentistry, “Victor Babeș” University of Medicine and Pharmacy, Eftimie Murgu Square 2, 300041 Timișoara, Romania; izabella.kovacs@umft.ro; 3 Department of Prosthodontics, TADERP Research Center, Faculty of Dentistry, “Victor Babeș” University of Medicine and Pharmacy, B-dul Revoluției 1989, No. 9, 300580 Timișoara, Romania; andrei.faur@umft.ro (A.-B.F.); jivanescu.anca@umft.ro (A.J.); 4 Department of Mechanics and Strength of Materials, Politehnica University Timișoara, 300006 Timișoara, Romania; liviu.marsavina@upt.ro

**Keywords:** 3D printing, 3D-printable dental resins, fracture resistance, Weibull analysis, fracture toughness, failure morphology, stereomicroscopy

## Abstract

**Background and Objectives**: Three-dimensional printing is increasingly used to fabricate dental restorations; however, limited evidence is available on the mechanical performance and fracture behavior of printable resin materials used for endocrown restorations. Fracture load alone may not fully describe material performance, particularly when brittle or defect-sensitive failure occurs. This in vitro study aimed to compare the fracture resistance, Weibull parameters, experimental Mode I fracture toughness parameter, and failure patterns of three 3D-printed resin materials used for endocrown restorations. **Materials and Methods**: Thirty anatomically identical molar replicas were produced from a single prepared tooth model and restored with endocrowns fabricated from NextDent C&B MFH (NextDent B.V., Soesterberg, The Netherlands), SprintRay Crown (SprintRay Inc., Los Angeles, CA, USA), and BEGO VarseoSmile Crown Plus (BEGO GmbH & Co. KG, Bremen, Germany) (*n* = 10/group). The restorations were cemented and subjected to compressive loading until fracture. Maximum fracture force values were analyzed using Welch’s ANOVA and Weibull statistics. In parallel, single-edge-notched bend (SENB) specimens were fabricated from the same materials and tested using an ASTM D5045-based configuration to calculate an experimental Mode I fracture toughness parameter. Representative fractured crowns and standardized specimens were examined using stereomicroscopy to assess visible failure morphology. **Results**: No statistically significant difference in maximum fracture force was found among the three materials (Welch’s ANOVA, *p* = 0.217). The mean fracture force values were 862.37 N for NextDent C&B MFH, 804.37 N for SprintRay Crown, and 699.43 N for BEGO VarseoSmile Crown Plus. In the complementary analyses, BEGO VarseoSmile Crown Plus showed the highest Weibull modulus (*m* = 9.36), indicating a narrower distribution of fracture values, and the highest mean experimental Mode I fracture toughness parameter (5.250 MPa·m^0.5^) under the present experimental conditions. Qualitative stereomicroscopic analysis revealed material-dependent visible failure patterns: SprintRay Crown exhibited more extensive fragmentation, whereas BEGO VarseoSmile Crown Plus showed a more defined visible fracture pattern with less secondary fragmentation. **Conclusions**: Fracture load alone was insufficient to characterize the mechanical behavior of the tested materials fully. Weibull parameters, the experimental fracture toughness parameter, and failure morphology provided complementary information and should be considered when evaluating 3D-printed resin materials for endocrown restorations.

## 1. Introduction

Restoring endodontically treated posterior teeth remains a biomechanical and clinical challenge, particularly when extensive coronal tooth structure has been lost. Traditionally, such teeth have often been restored using post-and-core systems followed by full-coverage crowns. However, this approach may require additional removal of radicular dentin and may increase the risk of procedural complications, particularly in teeth with narrow, curved, or anatomically complex canals [1,2]. With the development of adhesive dentistry and minimally invasive restorative concepts, endocrowns have been proposed as a conservative alternative for restoring severely damaged endodontically treated molars. Endocrowns are monoblock restorations that obtain retention from the pulp chamber and cervical margins, relying on adhesive cementation rather than intraradicular post retention [1,3,4]. 

The endocrown concept is particularly relevant for molars because the pulp chamber can provide a favorable macromechanical retention form while avoiding post-space preparation. Commonly described preparation principles include a butt-joint cervical margin, occlusal reduction, a relatively flat pulpal floor, rounded internal line angles, sealed radicular spaces, and internal divergence compatible with the placement of adhesive restorations [1,5]. These features aim to preserve residual tooth structure while providing sufficient restorative thickness and bonding surface. Previous clinical and systematic studies have reported favorable performance of endocrowns and have supported their use as a minimally invasive approach for restoring endodontically treated posterior teeth [1,6]. However, the fracture behavior of endocrowns remains influenced by the restorative material and the loading direction, with axial and lateral forces yielding different fracture resistance values and failure patterns [7].

Computer-aided design and computer-aided manufacturing have contributed substantially to the development of endocrown restorations, enabling monolithic restorations to be designed and fabricated from ceramic, hybrid ceramic, and resin-based CAD/CAM materials [2,6]. Much of the available evidence regarding endocrown fracture behavior has focused on conventionally processed or subtractively manufactured restorations, especially lithium disilicate, polymer-infiltrated ceramic networks, and CAD/CAM resin composites [2,6]. More recently, additive manufacturing has enabled the fabrication of printable resin-based one-piece endodontic crowns and endocrown-type restorations [8,9]. Three-dimensional printing may offer advantages such as digital reproducibility, cost-efficient production, reduced material waste compared with subtractive methods, and fabrication of multiple restorations from CAD files [9,10]. However, the fracture behavior of additively manufactured resin-based restorations remains less established than that of conventional milled CAD/CAM materials, particularly when used in endocrown configurations [9,10].

Printable resin-based restorative materials differ in composition, filler content, polymer matrix, post-processing protocol, and manufacturer-indicated clinical use [11,12,13]. These differences may influence not only the maximum force required to fracture a restoration, but also the failure consistency, crack-growth resistance, and the resulting fracture pattern [11,12,13]. 

Because printable crown-and-bridge resins differ in manufacturer-stated indications and formulations, the present study included one long-term provisional micro-filled hybrid crown-and-bridge resin and two definitive crown restorative resins [14,15,16]. This selection enabled a standardized laboratory comparison of whether differences in manufacturer indication and material formulation are reflected in fracture resistance, Weibull parameters, fracture toughness, and visible failure morphology, without implying that the provisional material should be considered a definitive endocrown material.

Accordingly, the mechanical evaluation of these materials should consider not only maximum fracture force, which estimates load-bearing capacity under standardized laboratory conditions, but also complementary parameters describing failure variability and crack-extension resistance [7,17,18]. Weibull analysis describes the distribution of fracture data and is useful for evaluating the variability of brittle or defect-sensitive dental materials. A higher Weibull modulus indicates a narrower distribution of fracture values under the tested conditions, but should not be interpreted as a direct predictor of clinical longevity [18,19,20]. Fracture toughness evaluates resistance to crack extension from a defined defect or notch and is mechanically distinct from the maximum fracture force recorded for a complete restoration [21,22]. ASTM D5045-based single-edge-notched bend testing provides a controlled framework for assessing Mode I fracture toughness-related parameters in polymer-based materials, provided that the relevant validity requirements are fulfilled [23]. Together with qualitative stereomicroscopic assessment of visible failure morphology, these methods may help explain why materials with comparable fracture loads exhibit different failure patterns or fragmentation severity [24,25].

Therefore, the aim of this in vitro study was to compare the fracture resistance, Weibull parameters, experimental Mode I fracture toughness parameter, and stereomicroscopic failure patterns of three 3D-printable resin materials used for endocrown restorations: NextDent C&B MFH, SprintRay Crown, and BEGO VarseoSmile Crown Plus. The null hypothesis was that there would be no statistically significant difference among the tested materials in maximum fracture force. Weibull parameters, the experimental Mode I fracture toughness parameter, and failure morphology were evaluated as complementary outcomes.

## 2. Materials and Methods

### 2.1. Study Design and Ethical Approval

This in vitro study was designed to compare the fracture resistance, Weibull parameters, experimental Mode I fracture toughness parameter, and stereomicroscopic failure patterns of three 3D-printable resin materials used for endocrown restorations. A single extracted human maxillary molar was prepared and scanned; 30 identical replicas were 3D-printed and used as standardized substrates, thus minimizing anatomical variability between samples.

The study was conducted in accordance with the Declaration of Helsinki and was approved by the Scientific Research Ethics Committee of the “Victor Babeș” University of Medicine and Pharmacy, Timișoara, Romania, under Approval No. 49/28.05.2026.

Three commercially available 3D-printable resin materials were investigated: NextDent C&B MFH (NextDent B.V., Soesterberg, The Netherlands), SprintRay Crown (SprintRay Inc., Los Angeles, CA, USA), and BEGO VarseoSmile Crown Plus (BEGO GmbH & Co. KG, Bremen, Germany).

These materials differed in manufacturer-stated indication. NextDent C&B MFH is described by the manufacturer as a micro-filled hybrid 3D-print resin for 3D-printed crowns and bridges for long-term temporary use. SprintRay Crown is indicated for definitive full and partial crowns, whereas BEGO VarseoSmile Crown Plus is indicated for permanent single crowns, inlays, onlays, and veneers [14,15,16]. These materials were selected because they were commercially available, compatible with the digital manufacturing workflow used in the present study, and allowed comparison of materials with different manufacturer-stated indications and formulations. Therefore, NextDent C&B MFH was included as a commercially relevant long-term provisional crown-and-bridge resin comparator, not as a definitive endocrown material. 

The specimens were divided into three groups according to the endocrown material used: Group 1, NextDent C&B MFH; Group 2, SprintRay Crown; and Group 3, BEGO VarseoSmile Crown Plus. For clarity, the terms Set I, Set II, and Set III, as displayed in some figures, correspond respectively to Group 1 (NextDent C&B MFH), Group 2 (SprintRay Crown), and Group 3 (BEGO VarseoSmile Crown Plus) in the manuscript. Each group included 10 endocrown restorations cemented onto standardized printed tooth replicas. An a priori sample-size calculation or power analysis was not performed. The sample size of 10 endocrown assemblies per material group was selected based on comparable in vitro fracture-resistance studies, the standardized nature of the experimental design, and the feasibility of specimen fabrication and mechanical testing. Specimens were allocated to the experimental groups according to the restorative material used. No computer-generated randomization sequence was applied; however, all tooth replicas were fabricated from the same reference preparation and were anatomically identical, thereby minimizing allocation-related anatomical variability.

### 2.2. Reference Tooth Preparation and Endodontic Treatment

One extracted human maxillary molar was selected as the reference tooth for the experimental model. The tooth was intact and free of caries, restorations, cracks, or structural defects. After debridement, the tooth was stored in distilled water until preparation.

An endodontic access cavity was prepared using high-speed round diamond burs under water cooling. Canal negotiation was performed using size 10 manual K-files (Kendo, VDW GmbH, Munich, Germany), and a glide path was created using a ProGlider file (Dentsply Sirona, Charlotte, NC, USA). Root canal preparation was performed using X1 and X2 files from the ProTaper Next rotary system (Dentsply Sirona, Charlotte, NC, USA), with the files operated according to the manufacturer’s recommended parameters of 300 rpm and 250 g·cm. During instrumentation, the canals were irrigated with 5.25% sodium hypochlorite and EDTA solution. After instrumentation, apical gauging was performed with manual K-files to verify the final apical preparation size before obturation.

Root canal obturation was performed using the single-cone technique with matching ProTaper Next X2 gutta-percha cones (Dentsply Sirona, Charlotte, NC, USA) and ADSEAL sealer (Meta Biomed, Cheongju, Republic of Korea). The gutta-percha cones were seared at the canal orifices using a heated electronic plugger (Woodpecker Fi-P, Woodpecker, Guilin, China) and compacted vertically. The canal orifices and pulp chamber floor were sealed with SDR flow+ Bulk Fill Flowable (Dentsply Sirona, Charlotte, NC, USA).

After root canal treatment, the coronal portion of the tooth was sectioned 3 mm above the cementoenamel junction under water cooling. A standardized endocrown preparation was performed, consisting of a flat butt-joint margin and an intracoronal extension into the pulp chamber. The intracoronal extension of the reference preparation was approximately 3 mm, as measured with a periodontal probe; the preparation geometry was subsequently verified digitally from the STL file in Exocad DentalCAD version 3.0 (Exocad GmbH, Darmstadt, Germany) and reproduced in all printed replicas. Using the same STL file, the maximum mesiodistal and buccopalatal coronal dimensions of the prepared reference molar were 9.02 mm and 11.19 mm, respectively. Internal undercuts were removed to provide a common path of insertion and to allow standardized seating of the restorations.

### 2.3. Digital Scanning and Fabrication of Standardized Tooth Replicas

The prepared reference molar was scanned using a Medit i700 intraoral scanner (Medit, Seoul, Republic of Korea), and a digital STL file of the preparation was generated. This file served as the reference geometry for both the fabrication of standardized tooth replicas and the design of the endocrown restorations.

**Figure 1:**
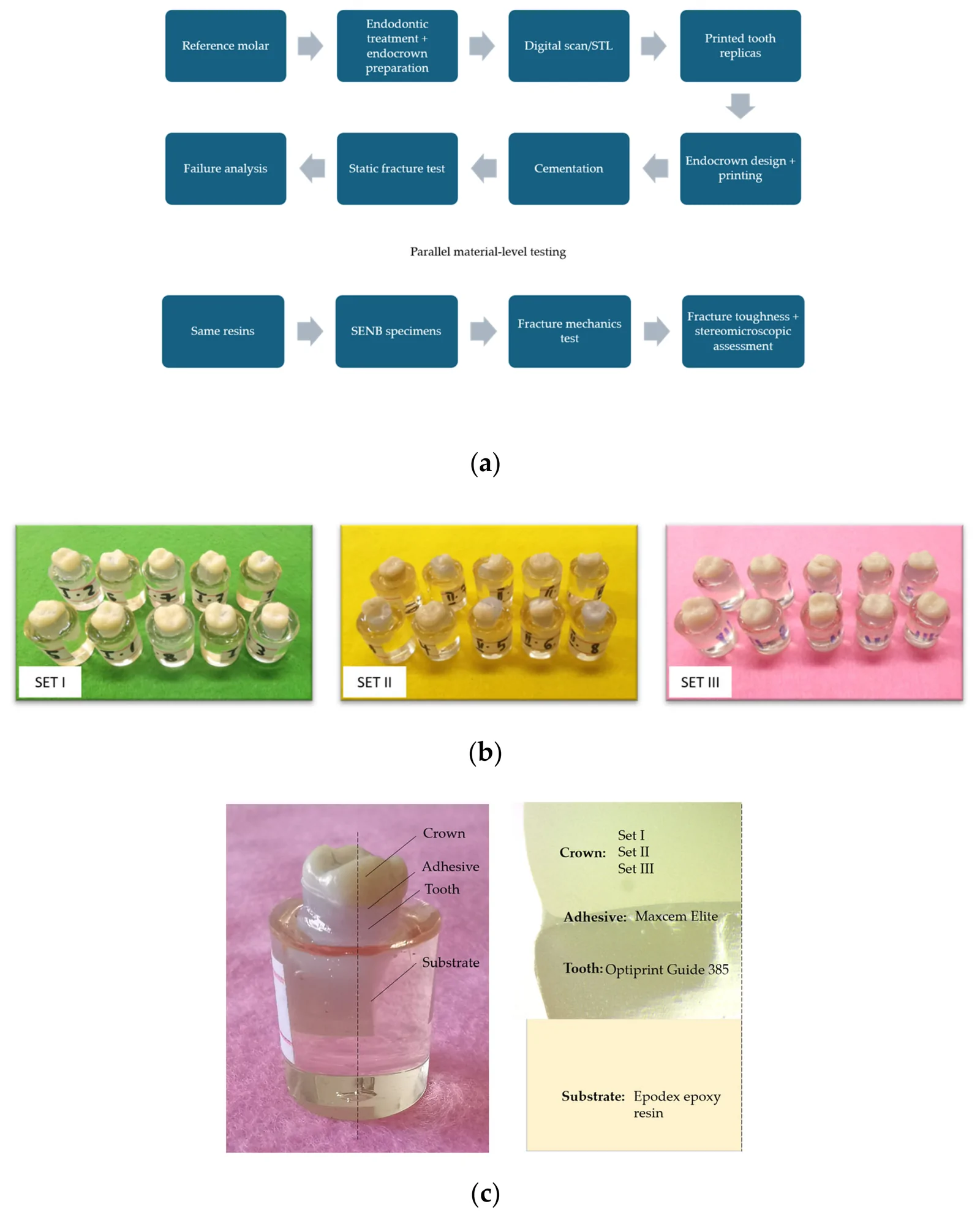
Experimental workflow and standardized endocrown specimen assembly. (**a**) Schematic overview of the endocrown fracture-resistance and SENB fracture-toughness workflows; (**b**) 3D-printed endocrown assemblies grouped according to material; (**c**) representative specimen showing the layered structure of the assembly: endocrown restoration, cement layer, printed tooth replica, and epoxy resin support.

Thirty anatomically identical tooth replicas were manufactured by additive manufacturing using Optiprint Guide resin (Dentona AG, Dortmund, Germany). The printed replicas were produced using a Prusa SL1 3D printer (Prusa Research, Prague, Czech Republic). After printing, the specimens were cleaned, post-processed, and visually inspected for printing defects, deformation, or incomplete polymerization. Only specimens with acceptable structural integrity and complete reproduction of the reference preparation were included in the study. The standardized endocrown assemblies and the representative layered specimen structure are shown in Figure 1.

Each printed tooth replica was embedded in an epoxy resin support (Epodex, Krefeld, Germany), leaving the prepared coronal portion exposed for restoration placement and subsequent mechanical testing. No periodontal ligament simulation was performed. The printed tooth replicas were embedded directly in epoxy resin to provide a rigid, standardized support and to isolate the restorative material as the main experimental variable.

### 2.4. Endocrown Design, Printing, and Post-Processing

The endocrown restorations were digitally designed using Exocad DentalCAD software, version 3.0. The same restoration design was used for all specimens in order to maintain identical external morphology, internal adaptation, occlusal anatomy, and restoration thickness across the three material groups. The same digital design also incorporated a standardized virtual cement gap of 0.08 mm, applied uniformly across all specimens as a controlled internal-fit parameter. The digital design was exported as STL files and used to fabricate the endocrowns via additive manufacturing.

To minimize variability and isolate the effect of restorative material, all other factors—reference preparation, digital design, scanner, printer, software, cementation procedure, and testing protocol—were standardized across all samples. The different experimental stages were standardized by assigning each stage to a single designated operator, who performed that stage consistently across all specimens and material groups. Endodontic treatment and endocrown preparation of the reference molar, as well as cementation of all restorations, were performed by the same experienced operator. Scanning and additive manufacturing procedures were performed by the same trained operator using the same workflow. Mechanical testing was performed by the same experienced engineer for all specimens.

The restorations were printed using masked stereolithography (MSLA), with a Prusa SL1 3D printer (Prusa Research, Prague, Czech Republic). Ten restorations were printed from each of the investigated materials: NextDent C&B MFH, SprintRay Crown, and BEGO VarseoSmile Crown Plus. All endocrown restorations were printed using the same build orientation, with support structures placed on the occlusal surface and the long axis of the restoration oriented approximately 90° to the build platform. The printing layer thickness was 0.05 mm (50 µm) for all three restorative materials.

Before fabrication of the study specimens, printer calibration and preliminary test prints were performed to verify adequate printing conditions; these test specimens were not included in the study. All specimens included in the study were printed in the same production session under the same printing conditions.

After printing, all endocrowns underwent identical post-processing. The restorations were cleaned in 99% isopropyl alcohol for 5 minutes to remove uncured resin, air-dried to remove residual solvent, and light post-cured using a Prusa CW1 washing, drying, and post-curing unit (Prusa Research, Prague, Czech Republic). Support structures were removed, and the intaglio surfaces were airborne-particle abraded with 35 µm aluminum oxide particles before cementation. This standardized protocol was applied uniformly to reduce workflow-related variability and maintain comparability among groups; it was not intended to compare material-specific manufacturer post-curing workflows.

### 2.5. Cementation Protocol

All endocrown restorations were cemented using Maxcem Elite self-adhesive resin cement (Kerr Corporation, Orange, CA, USA). After printing and post-processing, all restorations were visually inspected for visible printing defects, incomplete printing, support-related damage, or gross dimensional discrepancies. Before cementation, each restoration was checked for passive seating on its corresponding printed tooth replica. No advanced internal-defect analysis was performed before cementation. No final study specimens were excluded after inspection. Actual post-cementation cement film thickness was not measured; therefore, the 0.08 mm value refers to the virtual CAD cement-gap setting. The intaglio surface was cleaned with 99% ethanol, air-dried, and prepared for cementation according to the standardized protocol used in the present study.

Maxcem Elite was applied directly to the internal surface of each endocrown. The restoration was then seated onto the corresponding printed tooth replica using gentle, uniform manual pressure until complete seating was visually confirmed. All cementation procedures were performed by the same experienced operator using the same seating approach. No mechanical loading device was used; therefore, seating force was not quantified. Seating time was not quantified with a timer. A brief tack-curing phase was performed with an LED-E Woodpecker curing light (Woodpecker, Guilin, China) to facilitate the removal of excess cement. Excess cement was carefully removed from the margins, and final light-curing was performed for 10 s per axial surface. Where access to light was limited, chemical polymerization was allowed to continue according to the cement manufacturer’s recommendations.

Before fracture resistance testing, and following completion of the cementation protocol, all cemented endocrown assemblies were stored in distilled water at 37 °C for 24 h. This short standardized pre-test storage period was not intended to simulate artificial aging. No artificial aging procedure, such as thermocycling, prolonged water storage, cyclic loading, or mechanical fatigue, was performed before fracture resistance testing. This protocol was selected to compare the initial mechanical behavior of the three restorative materials under standardized short-term laboratory conditions.

### 2.6. Fracture Resistance Testing of Cemented Endocrowns

Fracture resistance testing was performed using a universal testing machine (LBG TC100, LBG, Azzano San Paolo, Italy). Each cemented endocrown assembly was positioned on a flat support surface, and compressive loading was applied to the occlusal surface using a 5 mm-diameter spherical metal indenter. The indenter was aligned with the longitudinal axis of the specimen and centered in the occlusal groove. Mechanical testing was performed under standardized laboratory conditions (23 ± 3 °C and 50 ± 10% relative humidity).

Axial loading with a 5 mm spherical indenter was selected to create a reproducible centralized occlusal contact and to allow standardized comparison of maximum compressive load-bearing capacity among groups under controlled laboratory conditions.

The specimens were loaded in compression at a crosshead speed of 5 mm/min until failure. The maximum force recorded before fracture was defined as the fracture resistance value and was expressed in Newtons (N). The fracture force data were subsequently used for statistical comparison and Weibull analysis. The operator performing mechanical testing was blinded to the material identity corresponding to each specimen group; maximum fracture force was recorded directly from the testing machine output.

### 2.7. Fracture Toughness Specimen Fabrication and Testing

**Figure 2:**
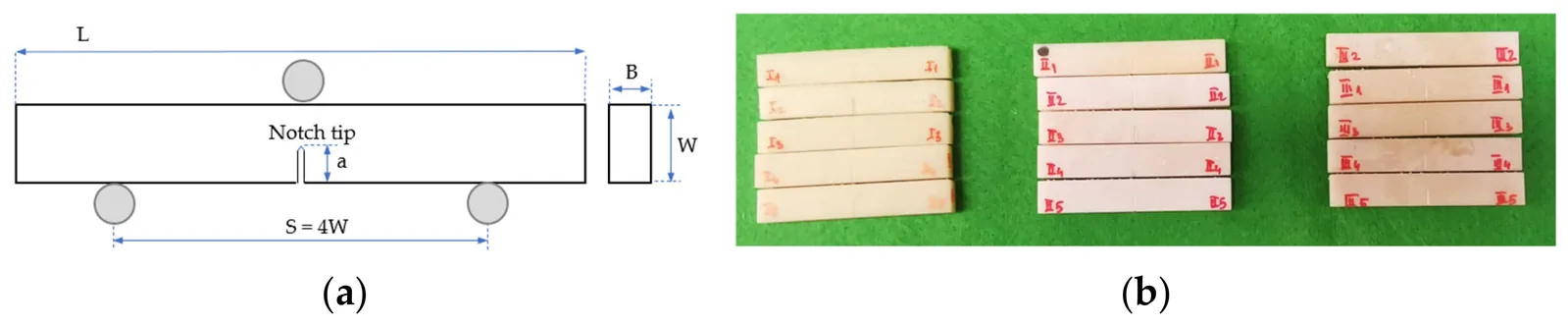
Specimen geometry and printed specimens used for experimental Mode I fracture toughness parameter testing: (**a**) Schematic representation of the ASTM D5045-based specimen geometry and three-point bending configuration; (**b**) printed single-edge-notched bend (SENB) specimens. L = specimen length; W = specimen width; B = specimen thickness; a = notch depth; S = support span.

In parallel with the endocrown fracture resistance experiment, standardized single-edge-notched bend specimens were fabricated from the same three resin materials in order to calculate an experimental Mode I fracture toughness parameter. The specimens were digitally designed with a predefined notch geometry and printed using the same materials investigated in the endocrown groups. Each SENB specimen had a length of 60 mm, a width of 10 mm, and a thickness of 4.5 mm. The predefined notch had a depth of 5.0 mm and a width of 0.4 mm. The three-point bending support span was 40 mm. Five specimens were produced for each material group (*n* = 5), resulting in a total of 15 SENB specimens. This number was selected to provide replicate measurements, considering the ASTM D5045-based testing configuration, material availability, and experimental feasibility. The notch was produced during additive manufacturing according to the predefined digital geometry. Before testing, the notch tip was manually sharpened using a sharp blade to reduce the notch-tip radius and provide a crack-like starting condition. No fatigue pre-cracking procedure was performed. The specimen geometry and printed single-edge-notched bend specimens are shown in Figure 2.

**Figure 3:**
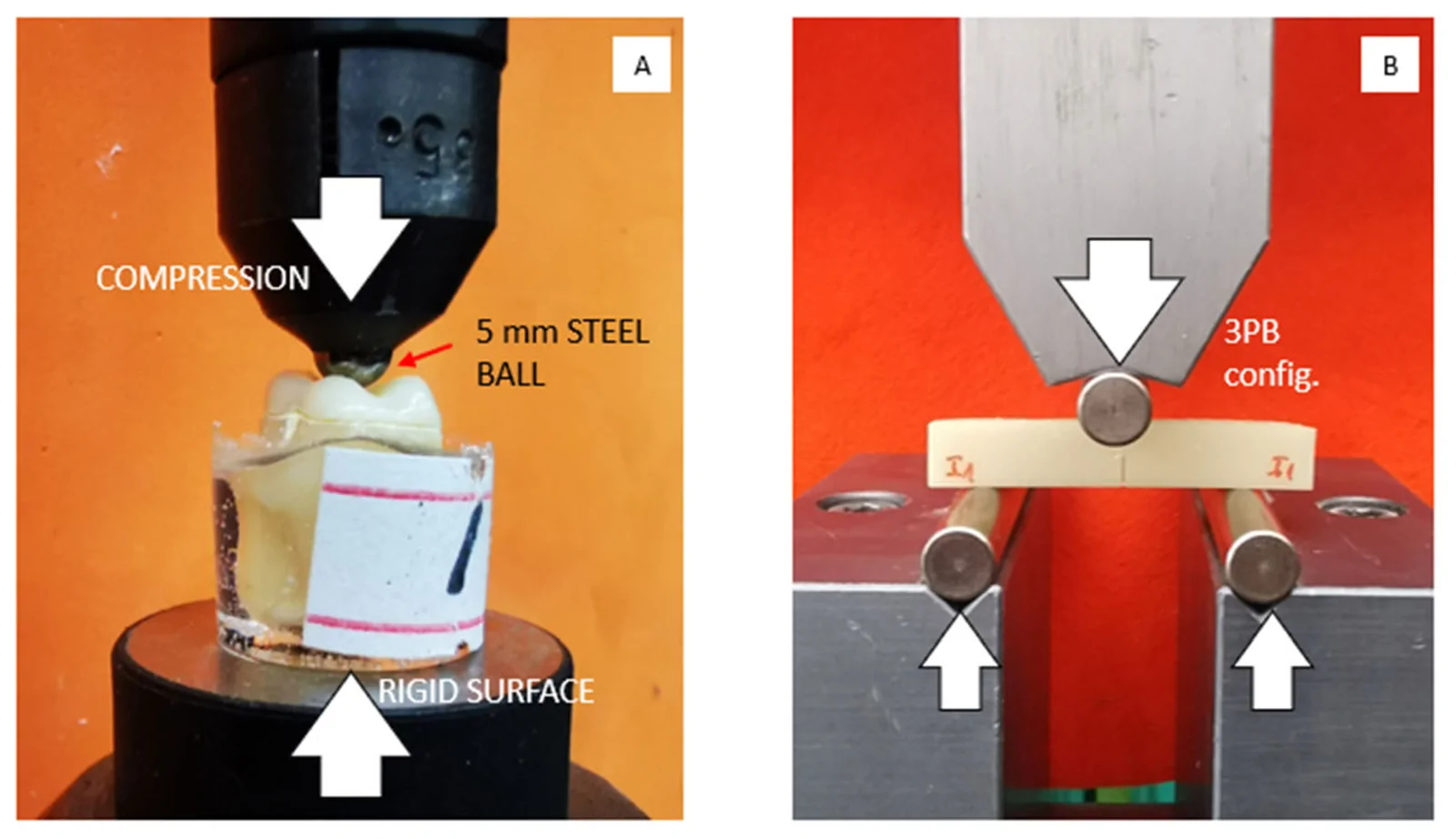
Mechanical testing configurations: (**A**) Compressive loading of the cemented endocrown assembly using a 5 mm spherical metal indenter centered on the occlusal surface; (**B**) three-point bending setup used for experimental single-edge-notched bend fracture toughness testing.

Fracture toughness testing was performed on a Zwick/Roell testing machine (Zwick-Roell GmbH & Co. KG, Ulm, Germany) at room temperature using an ASTM D5045-14 (2022)-based three-point bending configuration. A constant crosshead speed of 5 mm/min was applied until fracture. The mechanical testing configurations used for fracture resistance and fracture toughness testing are shown in Figure 3. 

Force-displacement data were recorded and used to calculate an experimental Mode I fracture toughness parameter.

### 2.8. Stereomicroscopic Failure Analysis

After mechanical testing, the fractured endocrown assemblies were inspected using a stereomicroscope (Optika SLX-3, OPTIKA S.r.l., Ponteranica, Italy) at 20× magnification. The analysis was performed qualitatively and was limited to external, visible fracture features, including dominant fracture lines, fragmentation pattern, and secondary crack branching. Representative fractured single-edge-notched bend specimens were also examined descriptively. No predefined scoring system or inferential comparison of failure modes was applied. Because neither scanning electron microscopy (SEM) nor formal fractographic analysis was performed, the stereomicroscopic observations were not used to determine true crack origin, fracture mirror areas, hackle marks, arrest lines, internal defects, or underlying fracture mechanisms.

The qualitative stereomicroscopic assessment was performed by an operator blinded to the material identity corresponding to each specimen group. The observations were interpreted descriptively and were not used for inferential comparison among groups.

### 2.9. Statistical Analysis

Data were analyzed using IBM SPSS Statistics version 26 (IBM Corp., Armonk, NY, USA). Data normality and homogeneity of variance were assessed using the Shapiro–Wilk and Levene’s tests, respectively. Because Levene’s test indicated unequal variances, Welch’s ANOVA was used to compare fracture resistance among the three material groups. Statistical significance was established at *α* = 0.05. As the overall Welch’s ANOVA was not statistically significant, no post hoc pairwise comparisons were performed.

Weibull analysis was performed using the fracture force values recorded for the cemented endocrown assemblies. A two-parameter Weibull distribution was fitted using the linearized graphical method. Failure probabilities were estimated using Bernard’s median-rank approximation *P_i_* = (*I* − 0.3)/(*n* + 0.4). The Weibull modulus (*m*), characteristic fracture force (*Fc*), and 95% confidence interval for the Weibull modulus were calculated from the fitted regression. Linear least-squares regression was performed using Microsoft Excel (Microsoft Corp., Redmond, WA, USA). The goodness of fit was evaluated using the coefficient of determination (*R*^2^).

Experimental Mode I fracture toughness parameters were calculated and reported descriptively as mean values for each material group. Because only five SENB specimens were tested per material and no fatigue pre-cracking procedure was performed, these results were intended to provide a descriptive comparison of crack-extension resistance under the present ASTM D5045-based SENB configuration and were not subjected to inferential statistical comparison.

## 3. Results

### 3.1. Fracture Resistance and Statistical Comparison

**Figure 4:**
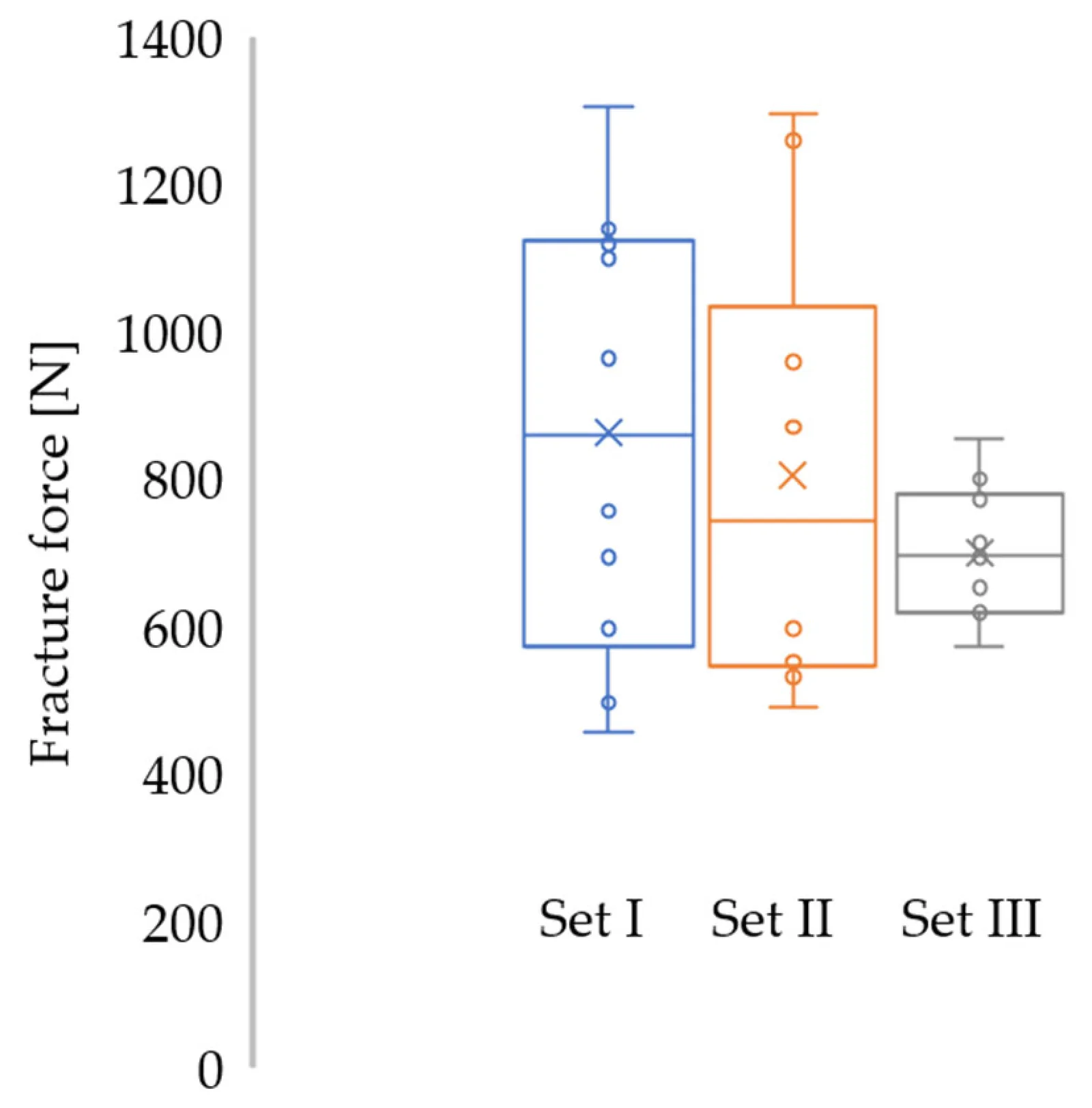
Fracture force values recorded for the three tested 3D-printable resin materials.

Fracture resistance testing was performed to determine the maximum load-bearing capacity of the cemented endocrown assemblies fabricated from the three investigated materials. The recorded fracture force values are shown in Figure 4.

The mean fracture force values were 862.37 N for Group 1, corresponding to NextDent C&B MFH, 804.37 N for Group 2, corresponding to SprintRay Crown, and 699.43 N for Group 3, corresponding to BEGO VarseoSmile Crown Plus.

The normality assumption was assessed using the Shapiro–Wilk test. No significant deviation from normal distribution was detected for Group 1 (*p* = 0.419), Group 2 (*p* = 0.101), or Group 3 (*p* = 0.846). However, homogeneity of variance was not satisfied according to Levene’s test (*p* = 0.001). Therefore, Welch’s ANOVA was applied and showed no statistically significant difference in maximum fracture force among the three groups (*F* = 1.711, *p* = 0.217).

### 3.2. Weibull Analysis of Fracture-Force Distribution

In addition to mean fracture force, Weibull analysis was performed to evaluate the distribution and variability of the fracture response in each material group. The descriptive fracture force values, 95% confidence intervals, Weibull parameters, and goodness-of-fit values are summarized in Table 1.

Group 1 and Group 2 showed similar Weibull moduli, with m values of 3.15 and 3.12, respectively. These values indicate greater scatter in fracture force values compared with Group 3. This interpretation is also supported by the higher coefficients of variation observed for these groups, which were 34.87% for Group 1 and 36.96% for Group 2.

In contrast, Group 3 showed the highest Weibull modulus (*m* = 9.36) and the lowest coefficient of variation (12.65%). Although this group exhibited the lowest mean and characteristic fracture forces, its higher Weibull modulus indicates a narrower distribution of fracture values. Therefore, under the present laboratory conditions, Group 3 showed the narrowest distribution of fracture values, whereas Groups 1 and 2 showed greater variability. However, this finding should be interpreted as a Weibull-based description of fracture-force scatter and not as evidence of overall material superiority.

**Figure 5:**
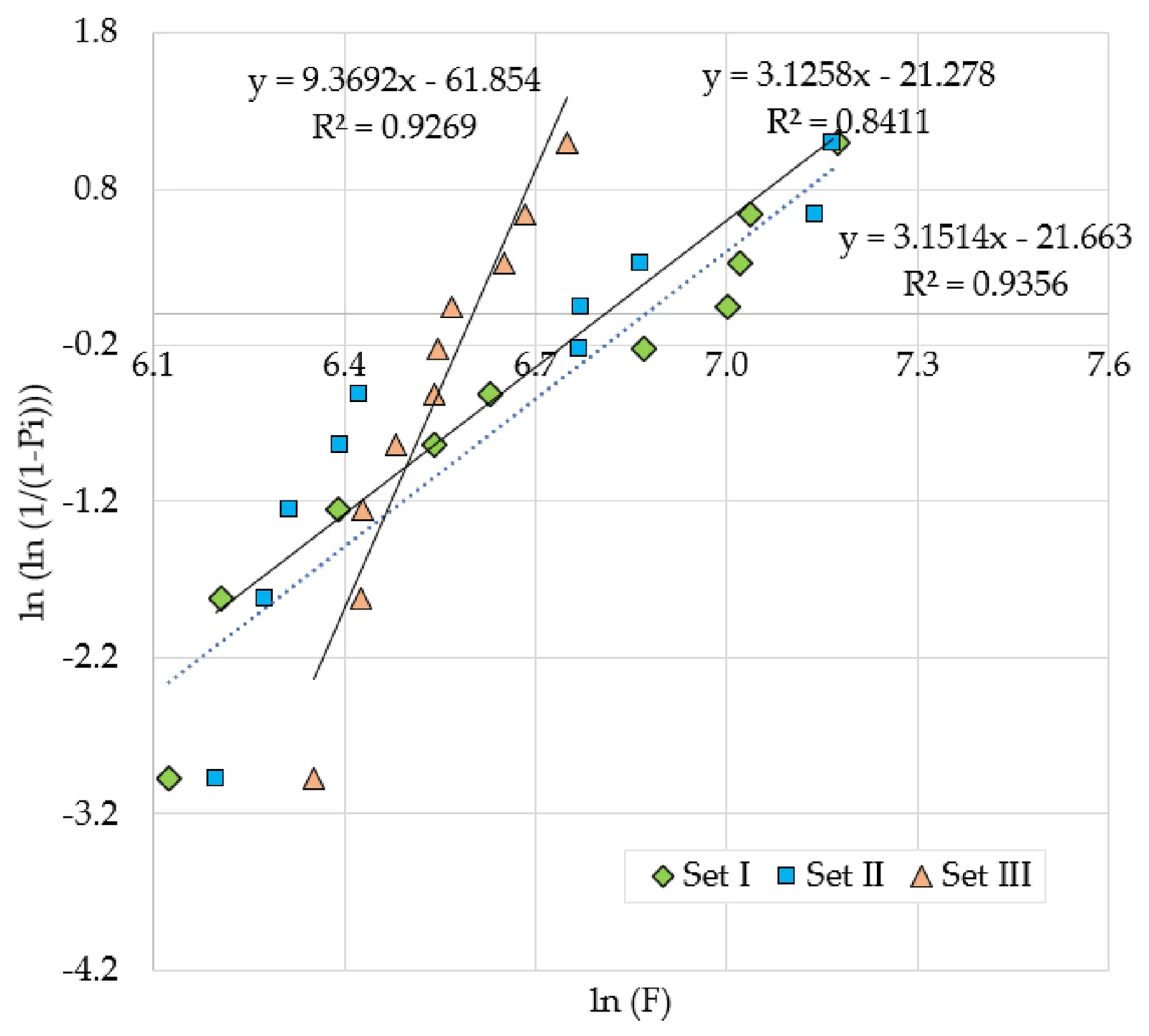
Weibull distribution of the three material groups.

The Weibull distributions of the three material groups are shown in Figure 5.

### 3.3. Stereomicroscopic Evaluation of Endocrown Failure Patterns

**Figure 6:**
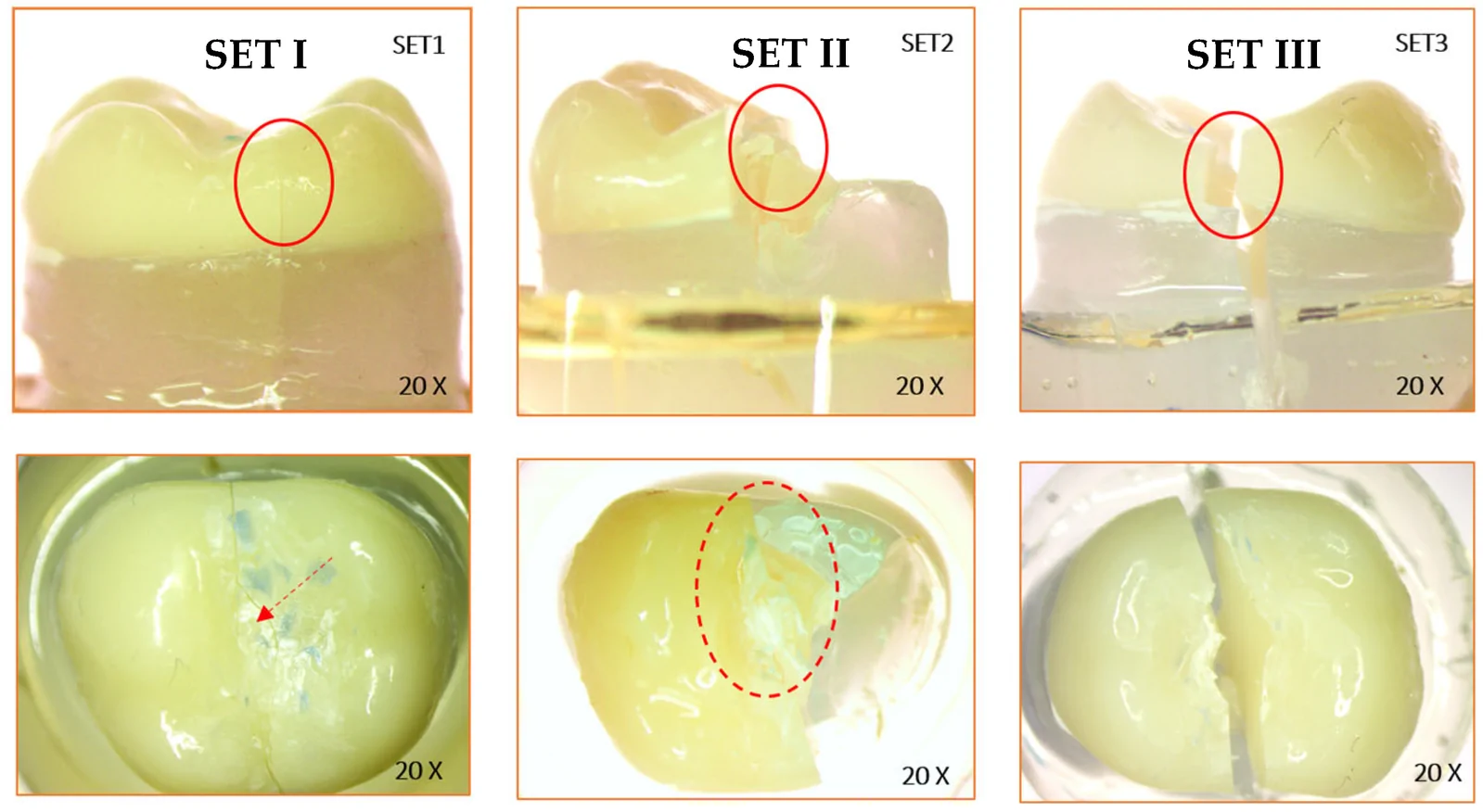
Stereomicroscopic observations of fractured endocrown assemblies. Red circles and the arrow highlight representative visible fracture features.

Qualitative stereomicroscopic examination revealed distinct visible failure morphologies among the three material groups, despite the absence of statistically significant differences in mean fracture force. Representative failure patterns are shown in Figure 6.

In Group 1, failure was characterized by a dominant visible fracture line extending from the occlusal loading area toward the cervical region. The crown remained largely intact, and the fracture pattern appeared relatively localized, with limited secondary branching and no extensive fragmentation.

Group 2 showed the most extensive visible fragmentation. Specimens from this group exhibited multiple fracture lines and separation of the crown into several fragments. The fractured areas appeared larger and more irregular than those observed in other groups.

In Group 3, failure generally followed a more defined visible fracture line through the crown thickness, resulting in separation into two major fragments without marked secondary cracking or material loss. The fracture surfaces appeared comparatively uniform, and the visible fracture pattern was more consistent than in Groups 1 and 2.

Overall, the stereomicroscopic observations provided descriptive information regarding external failure morphology. These findings should be interpreted as visible failure-pattern differences rather than as evidence of true crack origin, internal defects, or underlying fracture mechanisms.

### 3.4. Experimental Mode I Fracture Toughness Parameter and SENB Fracture Morphology

Experimental Mode I fracture toughness parameters were calculated using single-edge-notched bend specimens from the same three resin materials. The mean values obtained for each group are presented in Table 2.

Descriptively, Group 3 showed the highest mean experimental Mode I fracture toughness parameter (5.250 MPa·m^0.5^), followed by Group 2 (3.938 MPa·m^0.5^) and Group 1 (2.882 MPa·m^0.5^). Because no inferential statistical comparison was performed for the experimental fracture toughness parameter, these differences should be interpreted as descriptive trends rather than statistically supported differences among materials.

**Figure 7:**
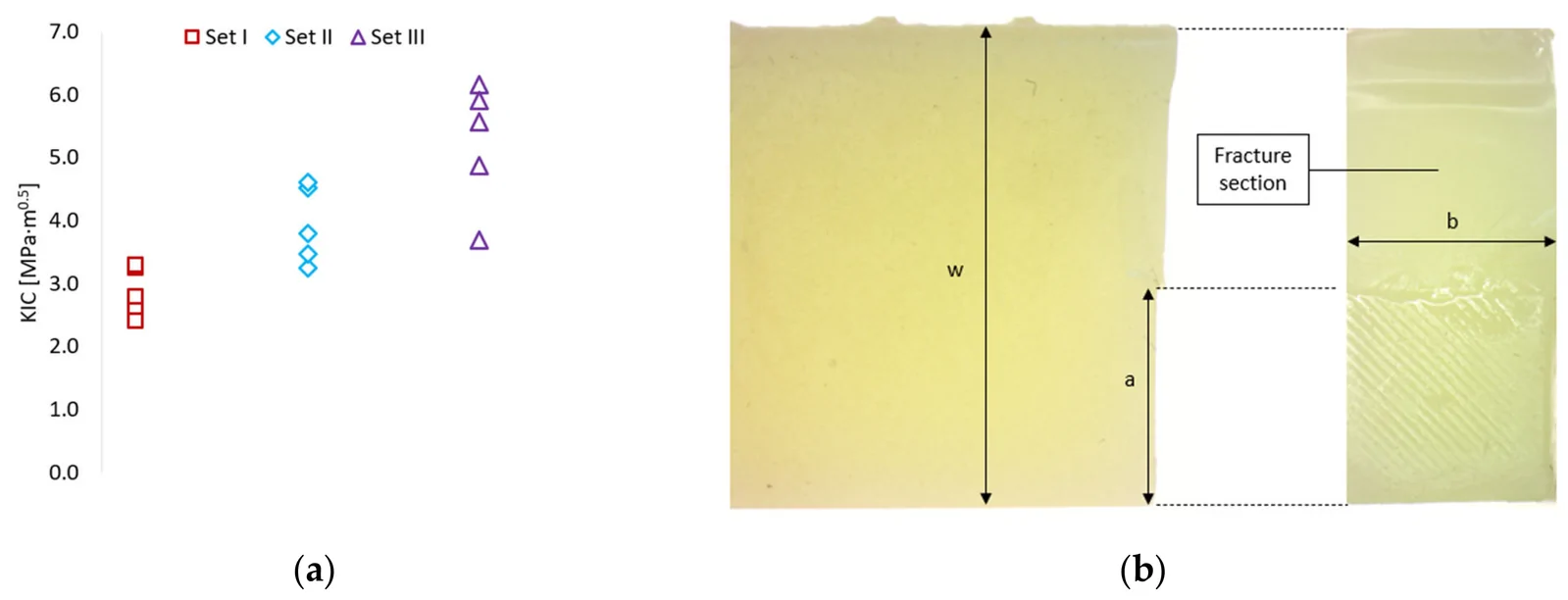
Experimental Mode I fracture toughness parameter and SENB fracture morphology. (**a**) Individual experimental fracture toughness observations for the three tested materials; (**b**) representative fractured SENB specimen.

The experimental fracture toughness results and representative single-edge-notched bend specimens after fracture are shown in Figure 7.

Descriptive examination of the fractured notched specimens showed a visible fracture line extending from the predefined notch region through the remaining ligament until complete separation occurred. The fracture surface appeared visually irregular near the notch region and more uniform along the remaining fracture plane. No marked secondary fracture lines or branching patterns were observed. These observations are consistent with the expected notch-guided fracture behavior of SENB specimens under three-point bending; however, because no SEM or formal fractographic analysis was performed, they should not be interpreted as definitive evidence of true crack origin or underlying fracture mechanisms. 

## 4. Discussion

The present in vitro study compared the fracture resistance, Weibull parameters, experimental Mode I fracture toughness parameter, and stereomicroscopic failure patterns of three 3D-printable materials used for endocrown restorations. The main finding was that the tested materials did not differ significantly in maximum fracture force. However, they showed different fracture-force distribution profiles, experimental fracture toughness parameter values, and failure morphologies. Therefore, the null hypothesis regarding maximum fracture force was not rejected. The Weibull parameters, experimental Mode I fracture toughness parameter, and failure morphology findings were interpreted as complementary outcomes.

In the present study, anatomical variability was minimized by using a single prepared maxillary molar as the reference model and by fabricating identical printed tooth replicas, thereby allowing the restorative material to be evaluated as the main experimental variable. Therefore, the experimental model should be interpreted as a standardized laboratory comparison of restorative materials, rather than as a direct simulation of clinical behavior in natural teeth.

Although mean fracture force differed numerically among the three groups, these differences were not statistically significant, indicating comparable load-bearing capacity under the static compressive loading conditions used in this study. The recorded fracture force values may be relevant to posterior loading; however, direct clinical extrapolation should be made with caution because maximum voluntary molar bite force varies substantially among individuals [26,27]. The absence of artificial aging beyond the standardized 24 h pre-test storage period was intended to maintain a controlled short-term comparison and to limit additional sources of variability. However, monotonic static fracture testing does not reproduce the cumulative degradation that occurs clinically through cyclic fatigue, thermal fluctuations, water aging, hydrolytic effects, periodontal ligament behavior, or complex multidirectional intraoral loading [28]. Therefore, the fracture force values reported in this study should be interpreted as short-term comparative laboratory outcomes rather than as predictors of long-term clinical performance. 

The Weibull analysis showed that Group 3 had the highest Weibull modulus and the lowest coefficient of variation, despite having the lowest mean fracture force. This indicates that BEGO VarseoSmile Crown Plus showed the narrowest distribution of fracture values under the present laboratory conditions. In contrast, Group 1 and Group 2 showed lower Weibull moduli and higher coefficients of variation, indicating greater scatter in fracture force and potentially greater sensitivity to defects. These findings emphasize that a higher mean fracture force does not necessarily correspond to lower variability in fracture response. Thus, Weibull parameters provide a laboratory-based description of fracture-force consistency under the tested conditions; however, in this non-aged in vitro model, they should be regarded as indicators of fracture-value distribution rather than direct predictors of long-term clinical survival. This interpretation is consistent with the use of the Weibull modulus as an indicator of strength-data dispersion in dental materials [7,18,19,20].

The qualitative stereomicroscopic observations provided additional descriptive information regarding the external failure patterns of the tested restorations. Rather than confirming true crack origin or underlying fracture mechanisms, these observations showed visible differences in fragmentation severity and fracture-line definition among groups. This descriptive information may be clinically relevant because extensive or catastrophic fragmentation may compromise repairability and remaining tooth structure, whereas less destructive fracture patterns are generally considered more favorable [7,17,25]. However, because no scanning electron microscopy (SEM) or formal fractographic analysis was performed, these findings should not be interpreted as definitive evidence of crack origin, internal defects, or underlying fracture mechanisms [19].

In the present study, Group 3 showed the highest mean experimental Mode I fracture toughness parameter, followed by Groups 2 and 1. Because a standardized fatigue pre-crack was not introduced and complete ASTM D5045 validity requirements for reporting strict KIC values could not be fully demonstrated, the obtained values should be considered experimental fracture toughness parameters rather than ASTM-valid KIC values. Therefore, these results should be interpreted primarily as descriptive material-level observations under the present ASTM D5045-based SENB testing configuration. In addition, because the fracture toughness data were not subjected to inferential statistical comparison, the higher mean value observed for Group 3 should not be interpreted as a statistically supported difference or as evidence of overall material superiority, especially because maximum fracture force did not differ significantly among the tested endocrown groups.

Overall, maximum fracture force alone did not fully characterize the behavior of the tested materials. The complementary outcomes showed differences in fracture-value distribution, mean experimental Mode I fracture toughness parameter, and visible failure behavior under the present laboratory conditions. However, these findings should not be interpreted as demonstrating overall material superiority, because maximum fracture force did not differ significantly among groups.

Recent studies evaluating additively manufactured one-piece endodontic crown or endocrown-type restorations have similarly emphasized that fracture behavior is influenced by material type, preparation design, margin configuration, pulp chamber depth, and aging conditions [8,9]. Bahadır et al. reported that different preparation designs of 3D-printed one-piece endodontic crowns produced comparable fracture resistance values but different fracture patterns after thermal aging [8]. Donmez et al. also showed that material type and geometric factors should be considered when interpreting the fracture strength of additively manufactured one-piece endodontic crowns fabricated from resins for definitive use [9]. Compared with these studies, the present investigation adds complementary material-level information by combining fracture resistance testing of cemented endocrown assemblies with Weibull analysis, experimental SENB fracture toughness testing, and qualitative stereomicroscopic assessment. However, direct comparison remains limited because recent studies on permanent 3D-printed endocrown-type restorations have mainly focused on fracture strength and failure patterns, whereas fracture toughness and Weibull parameters are less frequently reported. 

An important consideration is that, according to the manufacturer, NextDent C&B MFH is indicated for long-term temporary crowns and bridges. In contrast, SprintRay Crown and BEGO VarseoSmile Crown Plus are indicated for definitive or permanent restorations [14,15,16]. Therefore, the inclusion of NextDent C&B MFH should be interpreted as a laboratory comparison with a commercially available long-term provisional crown-and-bridge resin, rather than as an attempt to support its use as a definitive endocrown material. The high mean fracture force recorded for this material reflects its behavior under the present standardized test conditions and should not override the manufacturer’s clinical indication.

Although maximum fracture force did not differ significantly among groups, the observed differences in fracture-value distribution, mean experimental fracture toughness parameter, and visible failure morphology may be partly related to differences in resin composition, filler content, polymer matrix, filler–matrix interaction, degree of conversion, and post-processing response. Mechanical behavior may also be influenced by manufacturing and post-curing conditions. Beyond endocrown-specific fracture studies, broader investigations of additively manufactured crown-and-bridge resins have shown that resin formulation, filler content, printing workflow, aging, and post-curing conditions can influence flexural properties, wear behavior, fracture resistance, microhardness, degree of conversion, and color stability [11,12,13,29,30].

In the present study, identical post-processing conditions were applied to all materials to reduce workflow-related variability and to allow comparison under a standardized experimental protocol; however, this approach may not reproduce the optimal manufacturer-specific processing condition for each material. Although the solvent-cleaning steps recommended by the manufacturers are broadly comparable in purpose and duration, the manufacturers’ instructions differ in recommended post-curing devices, curing parameters, and finishing procedures [14,15,16]. Because post-curing and finishing conditions may influence polymerization, degree of conversion, residual monomer content, surface characteristics, and mechanical properties, the results should be interpreted in relation to the standardized post-processing protocol used in this study.

Because the present study did not include chemical, microstructural, filler-distribution, or degree-of-conversion analyses, the relationship between material composition and fracture behavior should be interpreted cautiously and remains an explanatory hypothesis rather than a demonstrated mechanism.

From a laboratory perspective, these findings indicate that the material with the highest mean fracture force may not necessarily show the narrowest fracture-value distribution or the highest mean experimental fracture toughness parameter. Under the present experimental conditions, Weibull analysis, experimental fracture toughness testing, and visible failure morphology provided complementary information beyond maximum fracture force alone. Clinically, this suggests that material selection for endocrown restorations should not be based solely on maximum fracture force, but should also consider consistency of fracture response, resistance to crack extension, and the potential repairability or severity of failure patterns [7,17,25]. Nevertheless, these findings should be interpreted as comparative laboratory outcomes and not as evidence of clinical superiority or long-term predictability of any tested material.

Several limitations should be considered when interpreting these findings. First, monotonic static compressive loading was used, and the specimens were tested without artificial aging; therefore, cyclic fatigue, thermomechanical aging, water aging, hydrolytic degradation, periodontal ligament damping, and oblique or lateral loading were not reproduced. Second, standardized 3D-printed tooth replicas fabricated from Optiprint Guide surgical-guide resin were used instead of natural teeth. This minimized anatomical variability and allowed the restorative material to be investigated as the main experimental variable, but the printed substrate should be regarded as a standardized experimental support rather than as a validated dentin or enamel analogue. Third, the sample size was limited for both the endocrown fracture-resistance and SENB fracture-toughness experiments, and experimental fracture toughness parameters were reported descriptively without inferential statistical comparison. Fourth, the use of a single standardized post-processing workflow improved comparability but did not allow independent evaluation of material-specific manufacturer workflows. In addition, the restorations were seated manually, and seating force was not quantified. Finally, failure morphology was evaluated qualitatively by stereomicroscopy only; no SEM, chemical, microstructural, or formal fractographic analyses were performed. Therefore, true crack origin, internal defects, and underlying fracture mechanisms could not be reliably identified.

Future studies should incorporate thermomechanical aging, more clinically representative loading conditions, and microstructural or SEM-based fractographic analyses to clarify fracture features and failure behavior. Clinical studies will ultimately be required to determine whether the differences observed in vitro translate into meaningful differences in long-term restoration performance. 

## 5. Conclusions

Within the limitations of this standardized in vitro model, no statistically significant difference in maximum fracture force was observed among the three tested 3D-printable resin materials under monotonic static compressive loading of specimens tested without artificial aging. However, differences were observed in the complementary Weibull parameters, mean experimental Mode I fracture toughness parameters, and failure morphology.

Among the complementary outcomes, NextDent C&B MFH showed limited fragmentation and the lowest mean experimental Mode I fracture toughness parameter, whereas SprintRay Crown showed an intermediate mean experimental Mode I fracture toughness parameter and exhibited the most extensive fragmentation. BEGO VarseoSmile Crown Plus showed the highest Weibull modulus, the lowest coefficient of variation, the highest mean experimental Mode I fracture toughness parameter, and a more defined visible fracture pattern under the present experimental conditions. However, these findings should be interpreted as complementary laboratory outcomes and not as evidence of overall superiority, because maximum fracture force did not differ significantly among the tested materials. 

These findings indicate that fracture load alone is insufficient to fully characterize the mechanical behavior of 3D-printed resin materials. Weibull analysis, experimental fracture toughness testing, and failure morphology evaluation provide complementary information and should be considered when assessing printable resin materials for endocrown restorations.

## Figures and Tables

**Table 1:** Descriptive fracture force values, Weibull parameters, and goodness-of-fit values.

Parameter	Group 1: NextDent C&B MFH	Group 2: SprintRay Crown	Group 3: BEGO VarseoSmile Crown Plus
Mean fracture force, N (95% CI)	862.37 (647.23–1077.49)	804.37 (591.72–1017.01)	699.43 (636.15–762.69)
Standard deviation (N)	300.73	297.26	88.44
Coefficient of variation (%)	34.87	36.96	12.65
Characteristic fracture force, *Fc* (N)	963.56	899.16	737.30
Weibull modulus, *m* (95% CI)	3.15 (2.47–3.82)	3.12 (2.01–4.23)	9.36 (7.22–11.51)
Coefficient of determination (*R*^2^)	0.93	0.84	0.92

**Table 2:** Mean experimental Mode I fracture toughness parameters.

Group	Material	Mean Experimental Mode I Fracture Toughness Parameter (MPa·m^0.5^)
Group 1	NextDent C&B MFH	2.882
Group 2	SprintRay Crown	3.938
Group 3	BEGO VarseoSmile Crown Plus	5.250

## Data Availability

The original contributions presented in this study are included in the article. Further inquiries can be directed to the corresponding authors.
